# Genomic Characterization of *hlyF*-positive Shiga Toxin–Producing *Escherichia coli*, Italy and the Netherlands, 2000–2019

**DOI:** 10.3201/eid2703.203110

**Published:** 2021-03

**Authors:** Federica Gigliucci, Angela H.A.M. van Hoek, Paola Chiani, Arnold Knijn, Fabio Minelli, Gaia Scavia, Eelco Franz, Stefano Morabito, Valeria Michelacci

**Affiliations:** Istituto Superiore di Sanità, Rome, Italy (F. Gigliucci, P. Chiani, A. Knijn, F. Minelli, G. Scavia, S. Morabito, V. Michelacci);; National Institute for Public Health and the Environment, Bilthoven, the Netherlands (A.H.A.M. van Hoek, E. Franz)

**Keywords:** Shiga toxin–producing *Escherichia coli*, extraintestinal pathogenic *E. coli*, hemolytic uremic syndrome, zoonoses, foodborne disease, antimicrobial resistance, whole-genome sequencing, *hlyF*, phylogenesis, Italy, the Netherlands, *Escherichia coli*, bacteria, enteric infections, bacterial zoonoses, food safety, STEC, ExPEC

## Abstract

Shiga toxin–producing *Escherichia coli* (STEC) O80:H2 has emerged in Europe as a cause of hemolytic uremic syndrome associated with bacteremia. STEC O80:H2 harbors the mosaic plasmid pR444_A, which combines several virulence genes, including *hlyF* and antimicrobial resistance genes. pR444_A is found in some extraintestinal pathogenic *E. coli* (ExPEC) strains. We identified and characterized 53 STEC strains with ExPEC-associated virulence genes isolated in Italy and the Netherlands during 2000–2019. The isolates belong to 2 major populations: 1 belongs to sequence type 301 and harbors diverse *stx_2_* subtypes, the intimin variant *eae-ξ*, and pO157-like and pR444_A plasmids; 1 consists of strains belonging to various sequence types, some of which lack the pO157 plasmid, the locus of enterocyte effacement, and the antimicrobial resistance–encoding region. Our results showed that STEC strains harboring ExPEC-associated virulence genes can include multiple serotypes and that the pR444_A plasmid can be acquired and mobilized by STEC strains.

Shiga toxin–producing *Escherichia coli* (STEC) is a group of enteric pathogens that cause foodborne disease ranging from uncomplicated diarrhea to hemorrhagic colitis (HC) or hemolytic uremic syndrome (HUS) (*1*). The most serious complication of STEC infection is HUS, which can be fatal.

STEC strains produce Shiga toxins (Stx), a family composed of 2 main types of cytotoxins: Stx1 and Stx2 (*2*). Stx1 is classified into subtypes a, c, and d; Stx2 is classified into subtypes a–k (*3*–*6*). Lysogenic bacteriophages harbor the genes for different types of Stx; infected bacteria then produce the protein (*7*). Although the production of Stx plays a central role in the pathogenesis of STEC-associated illness, the development of HC and HUS requires an efficient host colonization by the infecting STEC. Many HUS-associated STEC strains possess a chromosomal pathogenicity island, defined as the locus of enterocyte effacement (LEE), which is associated with the attaching and effacing lesions (*8*) described in enteropathogenic *E. coli* (*9*), or possess the genetic machinery conferring the enteroaggregative pattern of adhesion to the enterocyte described in enteroaggregative *E. coli* (*10*,*11*). Other STEC strains harbor colonization factors of enterotoxigenic *E. coli* (*12**,**13*) and genes encoding virulence features associated with extraintestinal pathogenic *E. coli* (ExPEC) (*14**,**15*). The ExPEC-associated virulence genes code for aerobactin (encoded by *iucC*), salmochelin (*iroN*), serum resistance protein (*iss*), a putative secretion system I (*etsABC*), omptin (*ompT*), hemolysin (*hlyF*), and bacteriocins (*cia* and *cva*) (*14*,*15*).

STEC strains belonging to the O157, O26, O103, O111, and O145 serogroups are considered critical public health concerns. Nevertheless, since 2015, other STEC serogroups have been increasingly associated with HUS and other infections in humans (*16*). Among these, O80 is emerging in Europe (*17*–*21*); since 2015, it has become a predominant serogroup associated with HUS in children in France (*22*). In addition to the typical clinical features of a STEC infection, bacteremia can develop in patients with STEC O80 (*19*,*23*).

Virulence genes increase the pathogenicity of STEC strains. For example, the strains associated with HUS are characterized by specific subtypes of the *stx*_2_ gene, mainly *stx*_2a_, *stx*_2c_, and *stx*_2d_ (*24*). In 2020, experts proposed a new approach to categorizing STEC infections on the basis of virulence genes (*24*). STEC O80 strains possess virulence genes carried by mobile genetic elements associated with intestinal and extraintestinal pathogenic *E. coli* (*14*). Such strains harbor the LEE locus, the *stx*_2_ gene, and a plasmid resembling the pO157 first described in STEC O157 serogroup carrying virulence genes including the enterohemolysin encoding gene (*ehxA*) (*25*,*26*). In addition, these strains often possess a peculiar mosaic plasmid called pR444_A. This pS88-like plasmid was first described in a STEC O80 strain isolated from a HUS patient with bacteremia in France (*14*). The pR444_A plasmid combines virulence genes of ExPEC strain S88, including the *hlyF*, *iro(BCDEN)*, *iss*, and *ompT* genes, with multiple antimicrobial resistance (AMR) determinants (*14**,**27**–**31*). The *hlyF* gene is associated with an increased production of outer membrane vesicles, possibly contributing to the release of cytolethal distending toxin and other chemicals involved in ExPEC pathogenesis (*32*).

Little data exist on the circulation of STEC strains harboring ExPEC-associated virulence traits. Infections from such pathogens rarely have been described outside France, except for 1 report about severe HUS caused by an O80:H2 strain in the Netherlands (*18*). We characterized the genomes of STEC strains with ExPEC-associated virulence traits isolated from infected patients and contaminated food in Italy and the Netherlands. We accessed these genomes through the Istituto Superiore di Sanità (Rome, Italy) and the National Institute for Public Health and the Environment (Bilthoven, the Netherlands). To infer population structure, we conducted a phylogenetic comparison of an additional 50 genomes of STEC strains with ExPEC-associated features from GenBank and RefSeq (https://www.ncbi.nlm.nih.gov/RefSeq).

## Material and Methods

### Bacterial Strains

For this study, we used STEC strains from the culture collections at the Istituto Superiore di Sanità and the National Institute for Public Health and the Environment. We investigated 500 STEC strains isolated in Italy during 2000–2019 by the National Reference Laboratory for *E. coli* as part of the national surveillance program for HUS and samples isolated from animal and food products in the framework of the official control activity. We also investigated 884 STEC strains isolated in the Netherlands from clinical samples collected during 2017–2019 as part of the surveillance for human STEC infections in the Netherlands.

### Whole-Genome Sequencing

We extracted the total DNA of the STEC strains from Italy from 2 mL of overnight culture of each strain grown in TSB at 37°C with the E.Z.N.A. Bacterial DNA kit (Omega Bio-tek, Inc., https://www.omegabiotek.com). We prepared sequencing libraries of ≈400 bp from 100 ng of total DNA using the NEBNext Fast DNA Fragmentation & Library Prep Set for Ion Torrent (New England BioLabs, https://www.neb.com). We amplified and enriched the libraries through emulsion PCR using the Ion OneTouch 2 System (Thermo Fisher Scientific, https://www.thermofisher.com) and sequenced on an Ion Torrent S5 platform (Thermo Fisher Scientific, https://www.thermofisher.com) using the ION 520/530 KIT-OT2 (Thermo Fisher Scientific) according to the manufacturer’s instructions for 400 bp DNA libraries on ION 530 chips.

We generated cell pellets of the STEC strains from the Netherlands using 1.8 mL of overnight culture of each strain grown in brain heart infusion broth (Thermo Fisher Scientific) at 37°C. We resuspended the pellets in DNA/RNA Shield (Zymo Research, https://www.zymoresearch.com) and sent them to BaseClear (https://www.baseclear.com) for DNA isolation and whole-genome sequencing. The BaseClear service generated paired-end 2 × 150 bp short-reads using a Nextera XT library preparation (Illumina, Inc., https://www.illumina.com) and sequenced the libraries on the HiSeq 2500 or NovaSeq 6000 systems (Illumina, Inc.). All the genomic sequences are available at the European Nucleotide Archive at the European Molecular Biology Laboratory (accession nos. PRJEB38068 and PRJEB38651).

### Bioinformatic Analysis

We conducted the bioinformatic analyses for the characterization of the genomes using the tools on the Galaxy public server ARIES (Istituto Superiore di Sanità, https://www.iss.it/site/aries) (A. Knijn, unpub. data, https://www.biorxiv.org/content/10.1101/2020.05.14.095901v1). We assembled the single-end reads from the Ion Torrent S5 platform using SPADES version 3.12.0 with default parameters (*33*) and filtered with the Filter SPAdes repeats tool (https://github.com/phac-nml/galaxy_tools) with default parameters to remove the contigs that were repeated or <1,000 bases. We trimmed the paired-end reads, filtered them with the Extended Randomized Numerical alignEr–filter (*34*), and assembled them de novo by using SPAdes version 3.10.0 (*33*).

### Basic Characterization of STEC Strains

We conducted multilocus sequence typing by using the MentaLiST tool version 0.2.3 (*35*), applying the scheme developed by Wirth et al. (*36*). We determined the virulence gene content of the STEC genomes and then identified the intimin gene (*eae*) subtype with the Patho_typing tool (https://github.com/B-UMMI/patho_typing) developed by the INNUENDO project (*37*) using the *E. coli* virulence genes database (*38*). We analyzed the assembled contigs with BLAST (https://blast.ncbi.nlm.nih.gov/Blast.cgi) and the blastn algorithm version 2.7.1. We determined the serotype by aligning the contigs with the reference sequences for the O and H antigen genes (*39*). We also used BLAST to identify the Stx subtype with the Statens Serum Institut Shiga toxin subtypes database (https://bitbucket.org/genomicepidemiology/virulencefinder_db/src/master/stx.fsa). We conducted phylogrouping using a blastn search of the specific genes (*40*) on the contigs.

### Characterization of STEC Strains Harboring ExPEC Virulence Genes

We used the *hlyF* gene as a putative marker for the pR444_A plasmid (*14*). We searched the assembled genomes for the *hlyF* gene (RefSeq accession no. NC_011980.1). We screened the *hlyF*-positive strains for antimicrobial and virulence genes associated with pR444_A using the ABRicate tool (https://github.com/tseemann/abricate).

We used PCR to confirm the presence of the *hlyF* gene in the strains from Italy, as described by Dissanayake et al. (*41*). We also investigated the presence of the pR444_A plasmid using the BRIG tool version 0.95 (http://brig.sourceforge.net) by aligning the contigs on the reference sequence from pR444_A (RefSeq accession no. NZ_QBDM01000004.1). In addition, we conducted the conjugation experiment among donor ED1284 and recipient CSH26Nal strains. We used streptomycin (10 µg/mL) as a selective agent for the pR444_A plasmid and nalidixic acid (10 µg/mL) for the recipient strain. We confirmed the colonies to be transconjugants with PCR selective for the *hlyF*, *traT*, *iroN*, *cvaC*, *iss*, and *ompT* genes. We also plated the colonies on Müller-Hinton agar plates containing trimethoprim (2 µg/mL), MacConkey plates to differentiate donor (*lac*+) and recipient (*lac*–) strains, and LB plates containing ampicillin (100 µg/mL), kanamycin (40 µg/mL), tetracycline (100 µg/mL), or sulfonamide (100 µg/mL).

### Cluster Analysis

To identify additional STEC strains with ExPEC-associated virulence features, we conducted a blastn search in GenBank and RefSeq for genomes positive for either *stx* (using the *stx*-subtypes sequence database) or *hlyF* (accession no. NC_011980.1) genes. We included these genomes in a cluster analysis along with the *hlyF*-positive STEC genomes produced in the current study. We carried out the analysis with core genome multilocus sequence typing (cgMLST) using the chewBBACA tool and the scheme developed by the INNUENDO project, which comprises 2,360 loci in total (*37*,*42*).

We considered the pairwise comparison to be reliable when >80% of loci were assigned to an allele. We calculated the distances between strains by pairwise comparison of the allelic profiles using the chewTree tool available on ARIES webserver (A. Knijn, unpub. data, https://www.biorxiv.org/content/10.1101/2020.05.14.095901v1). For each pair of samples, we excluded the alleles not found, only partially found, or not correctly assigned to any locus. We visualized the resulting dendrogram with FigTree version 1.4.4 (https://github.com/rambaut/figtree/releases).

## Results

### Circulating STEC Strains with ExPEC-Associated Virulence Genes 

The analyzed sequences had an average coverage of 118× and the assembled contigs an N50 average of 94,346 bp (Appendix 1 Table 1). Screening for the *hlyF* gene suggested the presence of the pR444_A plasmid in 53 (3.8%) of 1,384 STEC genomes (Appendix 1 Table 2). Of the 53 *hlyF*-positive strains, 30 had been isolated in Italy, mostly from patients with HUS or severe HC. Two were from food products of bovine origin in Italy (Appendix 1 Table 2). The remaining 23 STEC strains had been isolated from patients in the Netherlands, some of whom had diarrhea or bloody diarrhea and some of whom were hospitalized (Appendix 1 Table 2).

### Genomic Characterization of *hlyF*-Positive STEC Strains

The genomic analysis revealed that the 53 *hlyF*-positive STEC strains belonged to 10 different serotypes; O80:H2 was the most common (Appendix 1 Table 2). Most of the strains harbored the genes encoding the flagellar antigen H2, including 33 O80:H2 strains, 3 O186:H2 strains, and 4 O45:H2 strains (Appendix 1 Table 2). Two *hlyF*-positive STEC strains belonged to serotype O26:H11, one of the most common causes of HUS in Italy (*43*).

In silico MLST showed that ST301 was the most abundant sequence type (ST) (41/53; 77.4%) (Appendix 1 Table 2). Strains of 4 different serotypes belonged to ST301: 33 O80:H2 strains, 4 O45:H2 strains, 3 O186:H2 strains, and 1 O55:H9 strain. Two STEC O26:H11 strains belonged to ST21; the 10 remaining strains belonged to 6 other STs (Appendix 1 Table 2). All the strains belonged to the B1 phylogroup, except for 1 strain belonging to the B2 phylogroup and 3 strains whose phylogroup could not be identified (Appendix 1 Table 2).

In total, 49 strains tested positive for the *stx*_2_ gene and 4 for *stx*_1_ (Appendix 1 Table 2). The *stx_2_* gene subtyping revealed *stx*_2a_, *stx*_2b_, *stx*_2d_, *stx*_2e_, and *stx*_2f_ subtypes: *stx*_2a_ was the most common. All *stx*_1_ genes were *stx*_1a_ (Appendix 1 Table 2).

The 41 ST301 and 2 O26:H11 ST21 strains also harbored genes such as *ehxA* that are commonly found on pO157-like plasmids*.* In addition, they also tested positive for the intimin-coding *eae* gene, which indicates the presence of the LEE locus (Appendix 1 Table 2). All 41 ST301 genomes, regardless of serotype, carried the rare *eae-ξ* variant (Appendix 1 Table 2). The remaining 10 *hlyF*-positive strains tested negative for pO157-like plasmid genes and the LEE locus, except for strain NL1701358, which had the LEE locus with the *eae-λ3* variant. The NL1700566, NL1701474, NL1800025, and NL1800037 strains also carried the *hlyA* gene (data not shown), which encodes α-hemolysin (HlyA), a pore-forming toxin found in ExPEC strains that cause urinary tract infection (*44*,*45*).

**Figure 1:**
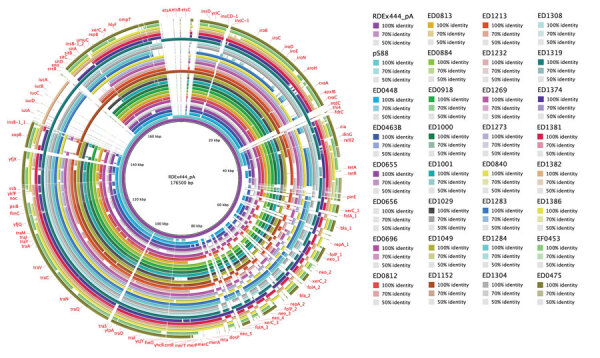
Whole-genome comparison of pR444_A–like plasmids in Shiga toxin–producing *Escherichia coli* strains harboring extraintestinal pathogenic *E. coli* (ExPEC)–associated virulence genes, Italy, 2000–2019. The pR444_A plasmid from RDEx444 strain was used as reference for alignment and gene annotation. Genomic annotation was performed by using the Prokka tool 1.14.5 (https://github.com/tseemann/prokka) and a multi-fasta file of trusted proteins related to ExPEC-associated genes on pR444_A. The comparative analysis also included the pS88 plasmid (GenBank accession no. CU928146.1) commonly found in ExPEC strains.

**Figure 2:**
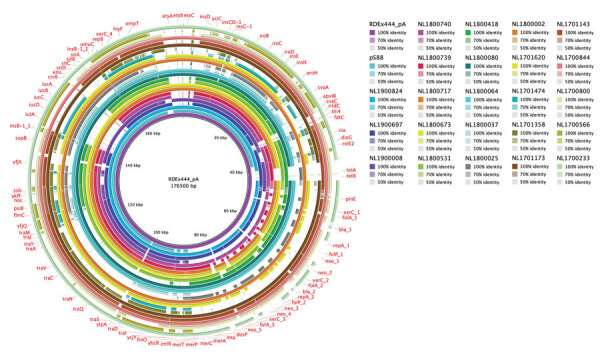
Whole-genome comparison of pR444_A–like plasmids in Shiga toxin–producing *Escherichia coli *strains harboring extraintestinal pathogenic *E. coli* (ExPEC)–associated virulence genes, the Netherlands, 2017–2019. The pR444_A plasmid from the RDEx444 strain was used as reference for alignment and gene annotation. Genomic annotation was performed with the Prokka tool 1.14.5 (https://github.com/tseemann/prokka) and a multi-fasta file of trusted proteins related to ExPEC-associated genes on pR444_A. The comparative analysis also included the pS88 plasmid (accession no. CU928146.1) commonly found in ExPEC strains.

 In addition to *hlyF*, the pR444_A plasmid also contains other virulence-associated genes such as *ompT*, *iss*, the *iroBCDEN* gene cluster, and a gene cassette that encodes determinants of AMR (*14*). The *hlyF*- positive STEC strains identified in this study carried many of these virulence determinants (Appendix 1 Table 2), suggesting the presence of a similar plasmid. Most *hlyF*-positive strains also had an AMR-encoding region (Appendix 1 Table 3). The alignment of the contigs on the pR444_A sequence further confirmed the presence of pR444_A–like plasmids in most *hlyF*-positive strains, regardless of country of isolation (Figures 1, 2). In most strains, we could not identify the regions of the pR444_A plasmid that harbor the *iucABCD* and *etsABC* genes. We conducted conjugation experiments to confirm the presence of a transferable pR444_A–like plasmid in the O26:H11 strain ED1284. After the mating, we observed that the *hlyF*, *iroN*, *cvaC*, *iss*, *traT*, and *ompT* genes were successfully transferred to the recipient K12 strain along with the cassette conferring resistance to streptomycin, ampicillin, sulfonamide, and trimethoprim.

### Phylogenetic Analysis of STEC Strains with ExPEC-Associated Virulence Genes

We conducted a whole-genome comparison; we included the STEC O80:H2 strain RDEx444 isolated in France (*14*) as reference strain, and 2 *hlyF*-negative STEC O80:H2 strains, ED0867 and ED1301, which were isolated in Italy, for comparative purposes. To more broadly analyze the population structure, we also included 50 *hlyF*-positive STEC strains retrieved from GenBank and RefSeq (Appendix 1 Table 4, 5). Then, we computed the number of allelic differences between strains (Appendix 2 Table).

**Figure 3:**
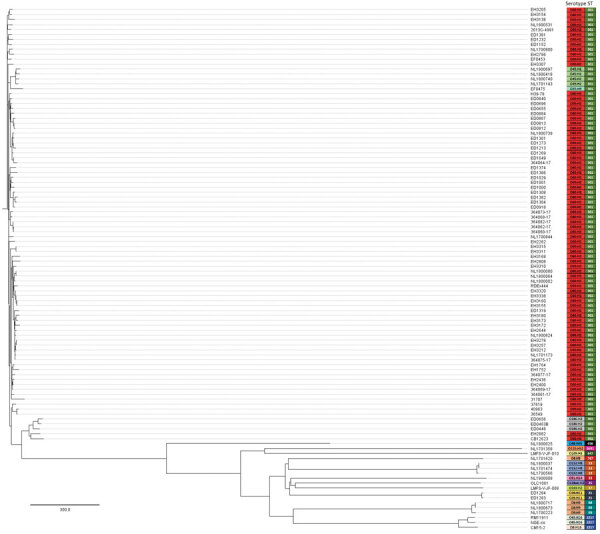
Cluster analysis by core genome multilocus sequence typing of Shiga toxin–producing *Escherichia coli* strains harboring extraintestinal pathogenic *E. coli*–associated virulence genes. The analysis also included the RDEx444 strain from France; 2 Shiga toxin–producing *E. coli* O80:H2 strains negative for the pR444_A plasmid (i.e., ED0867 and ED1301); and the set of 50 *E. coli* genomes positive either for *stx* or *hlyF* genes, downloaded from GenBank or RefSeq (www.ncbi.nlm.nih.gov/RefSeq). Each entry on the phylogenetic tree indicates the strain name, corresponding serotype, and sequence type. Colors indicates serotype and sequence type. Scale bar indicates the number of allelic differences.

The results of the cluster analysis clearly distinguished the strains belonging to ST301 (Figure 3). The strains belonging to serotype O80:H2 were related, showing a range of 2–210 allelic differences (Appendix 2 Table). The strains harboring the *stx_2d_* subtype, regardless of country origin, also were related (Figure 3). The branch containing the ST301 strains was divided into subclades corresponding to serotype (Figure 3). Among the ST301 strains, the O55:H9 EF0475 and O45:H2 strains were located close to the O80:H2 population, with a range of 58–219 allelic differences (Appendix 2 Table). The remaining genomes displayed >1,400 allelic differences from the ST301 strains (Appendix 2 Table).

## Discussion

*E. coli* bacteria continually acquire and lose genomic information carried by mobile genetic elements through horizontal gene transfer. This process contributes to the emergence of pathogenic *E. coli* variants. Horizontal gene transfer also can occur between pathogenic *E. coli* variants, producing hybrid pathogenic strains. Some STEC hybrid strains are highly virulent, such as enteroaggregative STEC serotype O104:H4, which caused one of the most severe STEC outbreaks ever reported (*46*).

Extraintestinal STEC serotype O80:H2 is a serious threat to public health. This hybrid clone was described in France in 2005 (*19*). Since then, extraintestinal STEC O80 strains have caused cases of severe HUS associated with bacteremia (*19*,*22*,*23*). In 2017, an O80:H2 strain caused a severe case of HUS with multiorgan failure in the Netherlands (*18*). Other cases of STEC O80:H2 infection have occurred in Switzerland and Belgium (*20*,*21*).

In this study, we demonstrated that genetic features associated with STEC and ExPEC strains are not restricted to the O80:H2 serotype. The STEC strains presenting ExPEC-associated virulence genes investigated in this study belonged to 10 different serotypes, with a high prevalence of O80:H2. We also identified 5 additional serotypes from the genomes available in GenBank and RefSeq. Most of the strains in this study, regardless of serogroup, were of ST301; had the flagellar antigen H2; and harbored the *stx*_2_, *eae-ξ*, and *ehxA* genes (Appendix 2 Table). This genetic homogeneity seems to extend beyond the presence of these genes; cgMLST showed that the ST301 genomes were related. The ST301 strains formed subclades corresponding to serotype and *stx* subtype (Figure 3). The *stx_2d_*–positive RDEx444 strain isolated in France in 2016 clustered with strains of the same *stx* subtype isolated in Italy and the Netherlands during 2016–2019, and in Belgium and Switzerland during 2015–2019, suggesting a spatiotemporal persistence of this clade in the last decade.

The phylogenetic analysis highlighted that the O80:H2, O45:H2, and O55:H9 genomes were closely related (Appendix 2 Table). These genomes also shared a clade with the 2 STEC O80:H2 strains that tested negative for pR444_A. This finding suggests that the pR444_A plasmid was acquired before these different serotypes diverged from a common ancestor of ST301. It is also possible that this plasmid was acquired in multiple events during the evolution of these serotypes; however, the presence of the rare *eae-ξ* gene in all these serotypes suggests that the plasmid was probably acquired in a single event.

The genomic analysis also revealed that the *hlyF*-positive STEC O26:H11 strains were distantly related to the other *hlyF*-positive STEC ST301 strains (Figure 3). These isolates resembled typical STEC O26:H11 strains because they possessed the *eae-β1* variant (Appendix 1 Table 2) and a pO157-like plasmid harboring the *katP* gene (not shown), which is not found on the pO157-like plasmid found in ST301 strains (*14*). STEC O26:H11 strain ED1284 successfully transferred the pR444_A plasmid through conjugation, indicating that STEC O26:H11 can acquire and maintain an additional large virulence plasmid conferring supplementary pathogenic potential while retaining the ability to spread this mobile genetic element to other *E. coli*. In Italy, we observed some HUS patients with STEC O80:H2 and enteropathogenic *E. coli* O26:H11 coinfection (S. Morabito, G. Scavia, unpub. data). Other O80:H2–O26:H11 coinfections were described during an outbreak linked to unpasteurized cheese (*47*), possibly explaining the presence of the pR444_A plasmid in STEC O26 strains.

In this study, 2 strains from Italy (Appendix 1 Table 2) and 1 strain from the GenBank and RefSeq databases were isolated from food products of bovine origin, suggesting the potential for zoonotic transmission. Since 1987, several studies have reported the isolation of STEC and atypical enteropathogenic *E. coli* with ExPEC-associated virulence genes from cattle (*21*,*48*). On the other hand, human infections caused by similar strains have been described only since 2008, mainly in the form of rare and mild disease (*21*). We showed that since 2001, STEC strains with ExPEC-associated virulence genes, especially those belonging to ST301, have caused many severe diseases including HUS, HC, and HC associated with severe diarrhea (Appendix 1 Table 2); these findings reinforce the high pathogenic potential of such hybrid strains.

Of the 53 *hlyF*-positive strains analyzed in this study, 4 also tested positive for the *hlyA* gene, which encodes an α-hemolysin typically produced by ExPEC strains that cause urinary tract infection (*44*,*45*). Such strains formed a distinct population of STEC strains; these strains lacked the pO157-like plasmid and the LEE locus and harbored a pR444_A plasmid without the AMR-encoding region (Figure 2; Appendix 1 Table 3). Accordingly, all their genomes grouped together in the cgMLST analysis and far from the bigger group of the ST301 strains (Figure 3; Appendix 2 Table).

In conclusion, STEC strains with ExPEC-associated virulence genes have circulated in Europe and caused human severe infections since 2001 or earlier. Moreover, we showed that this group of pathogenic *E. coli* includes multiple serotypes and sequence types. We propose that these strains belong to >2 different lineages that might have emerged after the dissemination of the ExPEC plasmid pR444_A into a heterogeneous population of STEC strains.

Appendix 1 Further information on *hlyF*-positive Shiga toxin–producing *Escherichia coli*, Italy and the Netherlands, 2000–2019.

Appendix 2 Further information on *hlyF*-positive Shiga toxin–producing Escherichia coli, Italy and the Netherlands, 2000–2019.

## References

[1] Karmali MA. Host and pathogen determinants of verocytotoxin-producing *Escherichia coli*-associated hemolytic uremic syndrome. Kidney Int Suppl. 2009;75:S4–7. 10.1038/ki.2008.608 19180132

[2] O’Brien AD, Tesh VL, Donohue-Rolfe A, Jackson MP, Olsnes S, Sandvig K, et al. Shiga toxin: biochemistry, genetics, mode of action, and role in pathogenesis. Curr Top Microbiol Immunol. 1992;180:65–94. 10.1007/978-3-642-77238-2_4 1324134

[3] Scheutz F, Teel LD, Beutin L, Piérard D, Buvens G, Karch H, et al. Multicenter evaluation of a sequence-based protocol for subtyping Shiga toxins and standardizing Stx nomenclature. J Clin Microbiol. 2012;50:2951–63. 10.1128/JCM.00860-12 22760050 PMC3421821

[4] Bai X, Fu S, Zhang J, Fan R, Xu Y, Sun H, et al. Identification and pathogenomic analysis of an *Escherichia coli* strain producing a novel Shiga toxin 2 subtype. Sci Rep. 2018;8:6756. 10.1038/s41598-018-25233-x 29712985 PMC5928088

[5] Lacher DW, Gangiredla J, Patel I, Elkins CA, Feng PC. Use of the *Escherichia coli* identification microarray for characterizing the health risks of Shiga toxin–producing *Escherichia coli* isolated from foods. J Food Prot. 2016;79:1656–62. 10.4315/0362-028X.JFP-16-176 28221838

[6] Yang X, Bai X, Zhang J, Sun H, Fu S, Fan R, et al. *Escherichia coli* strains producing a novel Shiga toxin 2 subtype circulate in China. Int J Med Microbiol. 2020;310:151377. 10.1016/j.ijmm.2019.151377 31757694

[7] O’Brien AD, Newland JW, Miller SF, Holmes RK, Smith HW, Formal SB. Shiga-like toxin-converting phages from *Escherichia coli* strains that cause hemorrhagic colitis or infantile diarrhea. Science. 1984;226:694–6. 10.1126/science.6387911 6387911

[8] Jerse AE, Yu J, Tall BD, Kaper JB. A genetic locus of enteropathogenic *Escherichia coli* necessary for the production of attaching and effacing lesions on tissue culture cells. Proc Natl Acad Sci U S A. 1990;87:7839–43. 10.1073/pnas.87.20.7839 2172966 PMC54845

[9] Nataro JP, Kaper JB. Diarrheagenic *Escherichia coli.* Clin Microbiol Rev. 1998;11:142–201. 10.1128/CMR.11.1.142 9457432 PMC121379

[10] Navarro-Garcia F. *Escherichia coli* O104:H4 pathogenesis: an enteroaggregative *E. coli*/Shiga toxin–producing *E. coli* explosive cocktail of high virulence. Microbiol Spectr. 2014;2. 10.1128/microbiolspec.EHEC-0008-2013 26104460

[11] Morabito S, Karch H, Mariani-Kurkdjian P, Schmidt H, Minelli F, Bingen E, et al. Enteroaggregative, Shiga toxin-producing *Escherichia coli* O111:H2 associated with an outbreak of hemolytic-uremic syndrome. J Clin Microbiol. 1998;36:840–2. 10.1128/JCM.36.3.840-842.1998 9508328 PMC104641

[12] Michelacci V, Maugliani A, Tozzoli R, Corteselli G, Chiani P, Minelli F, et al. Characterization of a novel plasmid encoding F4-like fimbriae present in a Shiga-toxin producing enterotoxigenic *Escherichia coli* isolated during the investigation on a case of hemolytic-uremic syndrome. Int J Med Microbiol. 2018;308:947–55. 10.1016/j.ijmm.2018.07.002 30030028

[13] Nyholm O, Halkilahti J, Wiklund G, Okeke U, Paulin L, Auvinen P, et al. Comparative genomics and characterization of hybrid Shigatoxigenic and enterotoxigenic *Escherichia coli* (STEC/ETEC) strains. PLoS One. 2015;10:e0135936. 10.1371/journal.pone.0135936 26313149 PMC4551483

[14] Cointe A, Birgy A, Mariani-Kurkdjian P, Liguori S, Courroux C, Blanco J, et al. Emerging multidrug-resistant hybrid pathotype Shiga toxin–producing *Escherichia coli* O80 and related strains of clonal complex 165, Europe. Emerg Infect Dis. 2018;24:2262–9. 10.3201/eid2412.180272 30457551 PMC6256387

[15] Malberg Tetzschner AM, Johnson JR, Johnston BD, Lund O, Scheutz F. *In silico* genotyping of *Escherichia coli* isolates for extraintestinal virulence genes by use of whole-genome sequencing data. J Clin Microbiol. 2020;58:e01269–20. 10.1128/JCM.01269-20 32669379 PMC7512150

[16] European Food Safety Authority and European Centre for Disease Prevention and Control (EFSA and ECDC). The European Union summary report on trends and sources of zoonoses, zoonotic agents and food-borne outbreaks in 2017. EFSA J. 2018;16:e05500. 32625785 10.2903/j.efsa.2018.5500 PMC7009540

[17] Nüesch-Inderbinen M, Cernela N, Wüthrich D, Egli A, Stephan R. Genetic characterization of Shiga toxin producing *Escherichia coli* belonging to the emerging hybrid pathotype O80:H2 isolated from humans 2010-2017 in Switzerland. Int J Med Microbiol. 2018;308:534–8. 10.1016/j.ijmm.2018.05.007 29884331

[18] Wijnsma KL, Schijvens AM, Rossen JWA, Kooistra-Smid AMDM, Schreuder MF, van de Kar NCAJ. Unusual severe case of hemolytic uremic syndrome due to Shiga toxin 2d-producing *E. coli* O80:H2. Pediatr Nephrol. 2017;32:1263–8. 10.1007/s00467-017-3642-3 28343354 PMC5440534

[19] Soysal N, Mariani-Kurkdjian P, Smail Y, Liguori S, Gouali M, Loukiadis E, et al. Enterohemorrhagic *Escherichia coli* hybrid pathotype O80:H2 as a new therapeutic challenge. Emerg Infect Dis. 2016;22:1604–12. 10.3201/eid2209.160304 27533474 PMC4994344

[20] Fierz L, Cernela N, Hauser E, Nüesch-Inderbinen M, Stephan R. Characteristics of Shigatoxin–producing *Escherichia coli* strains isolated during 2010–2014 from human infections in Switzerland. Front Microbiol. 2017;8:1471. 10.3389/fmicb.2017.01471 28824596 PMC5540938

[21] De Rauw K, Thiry D, Caljon B, Saulmont M, Mainil J, Pierard D. Characteristics of Shiga toxin producing– and enteropathogenic *Escherichia coli* of the emerging serotype O80:H2 isolated from humans and diarrhoeic calves in Belgium. Clin Microbiol Infect. 2019;25:111.e5–111.e8. 10.1016/j.cmi.2018.07.023 30076975

[22] Ingelbeen B, Bruyand M, Mariani-Kurkjian P, Le Hello S, Danis K, Sommen C, et al. Emerging Shiga-toxin-producing *Escherichia coli* serogroup O80 associated hemolytic and uremic syndrome in France, 2013-2016: Differences with other serogroups. PLoS One. 2018;13:e0207492. 10.1371/journal.pone.0207492 30419067 PMC6231688

[23] Mariani-Kurkdjian P, Lemaître C, Bidet P, Perez D, Boggini L, Kwon T, et al. Haemolytic-uraemic syndrome with bacteraemia caused by a new hybrid *Escherichia coli* pathotype. New Microbes New Infect. 2014;2:127–31. 10.1002/nmi2.49 25356358 PMC4184582

[24] Koutsoumanis K, Allende A, Alvarez-Ordonez A, Bover-Cid S, Chemaly M, et al.; EFSA BIOHAZ Panel. Scientific Opinion on the pathogenicity assessment of Shiga toxin–producing *Escherichia coli* (STEC) and the public health risk posed by contamination of food with STEC. EFSA Journal. 2020;18:5967.

[25] Karch H, Heesemann J, Laufs R, O’Brien AD, Tacket CO, Levine MM. A plasmid of enterohemorrhagic *Escherichia coli* O157:H7 is required for expression of a new fimbrial antigen and for adhesion to epithelial cells. Infect Immun. 1987;55:455–61. 10.1128/IAI.55.2.455-461.1987 2879796 PMC260350

[26] Beutin L, Prada J, Zimmermann S, Stephan R, Orskov I, Orskov F. Enterohemolysin, a new type of hemolysin produced by some strains of enteropathogenic *E. coli* (EPEC). Zentralbl Bakteriol Mikrobiol Hyg A. 1988;267:576–88. 10.1016/S0176-6724(88)80042-7 3289285

[27] Peigne C, Bidet P, Mahjoub-Messai F, Plainvert C, Barbe V, Médigue C, et al. The plasmid of *Escherichia coli* strain S88 (O45:K1:H7) that causes neonatal meningitis is closely related to avian pathogenic *E. coli* plasmids and is associated with high-level bacteremia in a neonatal rat meningitis model. Infect Immun. 2009;77:2272–84. 10.1128/IAI.01333-08 19307211 PMC2687354

[28] Morales C, Lee MD, Hofacre C, Maurer JJ. Detection of a novel virulence gene and a *Salmonella* virulence homologue among *Escherichia coli* isolated from broiler chickens. Foodborne Pathog Dis. 2004;1:160–5. 10.1089/fpd.2004.1.160 15992275

[29] Dozois CM, Daigle F, Curtiss R III. Identification of pathogen-specific and conserved genes expressed in vivo by an avian pathogenic *Escherichia coli* strain. Proc Natl Acad Sci U S A. 2003;100:247–52. 10.1073/pnas.232686799 12506201 PMC140941

[30] Chuba PJ, Leon MA, Banerjee A, Palchaudhuri S. Cloning and DNA sequence of plasmid determinant *iss*, coding for increased serum survival and surface exclusion, which has homology with lambda DNA. Mol Gen Genet. 1989;216:287–92. 10.1007/BF00334367 2546040

[31] Haiko J, Laakkonen L, Juuti K, Kalkkinen N, Korhonen TK. The omptins of *Yersinia pestis* and *Salmonella enterica* cleave the reactive center loop of plasminogen activator inhibitor 1. J Bacteriol. 2010;192:4553–61. 10.1128/JB.00458-10 20639337 PMC2937412

[32] Murase K, Martin P, Porcheron G, Houle S, Helloin E, Pénary M, et al. HlyF produced by extraintestinal pathogenic *Escherichia coli* is a virulence factor that regulates outer membrane vesicle biogenesis. J Infect Dis. 2016;213:856–65. 10.1093/infdis/jiv506 26494774

[33] Bankevich A, Nurk S, Antipov D, Gurevich AA, Dvorkin M, Kulikov AS, et al. SPAdes: a new genome assembly algorithm and its applications to single-cell sequencing. J Comput Biol. 2012;19:455–77. 10.1089/cmb.2012.0021 22506599 PMC3342519

[34] Del Fabbro C, Scalabrin S, Morgante M, Giorgi FM. An extensive evaluation of read trimming effects on Illumina NGS data analysis. PLoS One. 2013;8:e85024. 10.1371/journal.pone.0085024 24376861 PMC3871669

[35] Feijao P, Yao HT, Fornika D, Gardy J, Hsiao W, Chauve C, et al. MentaLiST - A fast MLST caller for large MLST schemes. Microb Genom. 2018;4. 10.1099/mgen.0.000146 29319471 PMC5857373

[36] Wirth T, Falush D, Lan R, Colles F, Mensa P, Wieler LH, et al. Sex and virulence in *Escherichia coli*: an evolutionary perspective. Mol Microbiol. 2006;60:1136–51. 10.1111/j.1365-2958.2006.05172.x 16689791 PMC1557465

[37] Llarena AK, Ribeiro-Gonçalves BF, Nuno Silva D, Halkilahti J, Machado MP, Da Silva MS, et al. INNUENDO: A cross‐sectoral platform for the integration of genomics in the surveillance of food‐borne pathogens. EFSA Supporting Publication. 2018;15:EN-1498. 10.2903/sp.efsa.2018.EN-1498

[38] Joensen KG, Scheutz F, Lund O, Hasman H, Kaas RS, Nielsen EM, et al. Real-time whole-genome sequencing for routine typing, surveillance, and outbreak detection of verotoxigenic *Escherichia coli.* J Clin Microbiol. 2014;52:1501–10. 10.1128/JCM.03617-13 24574290 PMC3993690

[39] Joensen KG, Tetzschner AM, Iguchi A, Aarestrup FM, Scheutz F. Rapid and easy in silico serotyping of *Escherichia coli* isolates by use of whole-genome sequencing data. J Clin Microbiol. 2015;53:2410–26. 10.1128/JCM.00008-15 25972421 PMC4508402

[40] Clermont O, Christenson JK, Denamur E, Gordon DM. The Clermont *Escherichia coli* phylo-typing method revisited: improvement of specificity and detection of new phylo-groups. Environ Microbiol Rep. 2013;5:58–65. 10.1111/1758-2229.12019 23757131

[41] Dissanayake DR, Octavia S, Lan R. Population structure and virulence content of avian pathogenic *Escherichia coli* isolated from outbreaks in Sri Lanka. Vet Microbiol. 2014;168:403–12. 10.1016/j.vetmic.2013.11.028 24388626

[42] Silva M, Machado MP, Silva DN, Rossi M, Moran-Gilad J, Santos S, et al. chewBBACA: A complete suite for gene-by-gene schema creation and strain identification. Microb Genom. 2018;4. 10.1099/mgen.0.000166 29543149 PMC5885018

[43] Scavia G, Gianviti A, Labriola V, Chiani P, Maugliani A, Michelacci V, et al. A case of haemolytic uraemic syndrome (HUS) revealed an outbreak of Shiga toxin-2-producing *Escherichia coli* O26:H11 infection in a nursery, with long-lasting shedders and person-to-person transmission, Italy 2015. J Med Microbiol. 2018;67:775–82. 10.1099/jmm.0.000738 29687765

[44] Goebel W, Hedgpeth J. Cloning and functional characterization of the plasmid-encoded hemolysin determinant of *Escherichia coli.* J Bacteriol. 1982;151:1290–8. 10.1128/JB.151.3.1290-1298.1982 7050085 PMC220407

[45] Dhakal BK, Mulvey MA. The UPEC pore-forming toxin α-hemolysin triggers proteolysis of host proteins to disrupt cell adhesion, inflammatory, and survival pathways. Cell Host Microbe. 2012;11:58–69. 10.1016/j.chom.2011.12.003 22264513 PMC3266558

[46] Frank C, Werber D, Cramer JP, Askar M, Faber M, an der Heiden M, et al.; HUS Investigation Team. Epidemic profile of Shiga-toxin-producing *Escherichia coli* O104:H4 outbreak in Germany. N Engl J Med. 2011;365:1771–80. 10.1056/NEJMoa1106483 21696328

[47] Espié E, Grimont F, Mariani-Kurkdjian P, Bouvet P, Haeghebaert S, Filliol I, et al. Surveillance of hemolytic uremic syndrome in children less than 15 years of age, a system to monitor O157 and non-O157 Shiga toxin-producing *Escherichia coli* infections in France, 1996-2006. Pediatr Infect Dis J. 2008;27:595–601. 10.1097/INF.0b013e31816a062f 18520972

[48] Blanco M, Blanco JE, Mora A, Dahbi G, Alonso MP, González EA, et al. Serotypes, virulence genes, and intimin types of Shiga toxin (verotoxin)-producing *Escherichia coli* isolates from cattle in Spain and identification of a new intimin variant gene (*eae*-xi). J Clin Microbiol. 2004;42:645–51. 10.1128/JCM.42.2.645-651.2004 14766831 PMC344521

