# Supplementary material for: Genomic Characterization of hlyF-positive Shiga Toxin–Producing Escherichia coli, Italy and the Netherlands, 2000–2019
Source: Emerg Infect Dis. 2021 Mar;27(3):853–61. doi: 10.3201/eid2703.203110 (PMC7920663; doi:10.3201/eid2703.203110)
Supplement: Appendix 1 — Further information on hlyF-positive Shiga toxin–producing Escherichia coli, Italy and the Netherlands, 2000–2019. [file 20-3110-Techapp-s1.pdf]

Article DOI: https://doi.org/10.3201/eid2703.203110

Genomic Characterization of *hlyF*-positive Shiga toxin–Producing *Escherichia coli*, Italy and the Netherlands, 2000–2019

Appendix 1

Appendix 1 Table 1. Genomic sequencing depth and assembly statistics of genomic sequences of *hlyF*-positive Shiga toxin–producing *Escherichia coli*, Italy and the Netherlands, 2000–2019

Strain	Sequencing depth*	Estimated contigs coverage†	No. contigs	N50
ED0448	95×	1.05×	177	123196
ED0463B	107×	1.05×	191	93269
ED0655	59×	1.06×	188	93733
ED0656	109×	1.08×	198	75535
ED0696	68×	1.05×	225	87928
ED0812	78×	1.09×	209	67677
ED0813	84×	1.07×	191	87938
ED0840	118×	1.09×	204	90781
ED0884	108×	1.1×	202	86312
ED0918	75×	1.06×	167	110175
ED1000	74×	1.08×	193	83903
ED1001	169×	1.1×	208	75640
ED1029	200×	1.08×	190	86691
ED1049	139×	1.08×	188	108440
ED1152	153×	1.09×	215	70160
ED1213	94×	1.09×	203	105660
ED1232	174×	1.06×	204	73316
ED1269	110×	1.08×	225	104490
ED1273	132×	1.09×	206	109870
ED1283	79×	1.09×	203	83131
ED1284	67×	1.09×	194	74252
ED1304	77×	1.09×	182	87927
ED1308	83×	1.06×	200	65716
ED1319	52×	1.07×	192	87931
ED1374	141×	1.05×	184	86314
ED1381	126×	1.06×	205	68640
ED1382	251×	1.08×	165	72686
ED1386	139×	1.05×	185	104629
EF0453	131×	1.06×	211	70199
EF0475	144×	1.06×	171	72821
NL1700223	101×	1.08×	438	143394
NL1700566	111×	1.08×	479	68240
NL1700800	63×	1.12×	759	93204
NL1700844	77×	1.11×	744	93004
NL1701143	63×	1.1×	720	91144
NL1701173	78×	1.08×	752	87756
NL1701358	85×	1.02×	302	209789
NL1701474	66×	1.06×	464	79826
NL1701620	111×	1.03×	327	106777
NL1800002	86×	1.11×	769	74413
NL1800025	107×	1.02×	405	66939
NL1800037	98×	1.06×	447	71815
NL1800064	168×	1.09×	792	72748
NL1800080	204×	1.09×	849	72727
NL1800418	89×	1.11×	709	92328
NL1800531	93×	1.13×	836	72973
NL1800673	112×	1.05×	338	190511
NL1800717	106×	1.06×	510	190511
NL1800739	138×	1.11×	750	91090

Strain	Sequencing depth*	Estimated contigs coverage†	No. contigs	N50
NL1800740	146×	1.09×	692	91999
NL1900008	95×	1.11×	359	134460
NL1900697	410×	1.11×	461	101567
NL1900824	193×	1.11×	510	94172

*Sequencing depth value indicates the total length of all reads divided by the estimated length of the *E. coli* genome (i.e., 5 Mb).
†Estimated contigs coverage is calculated by dividing the number of assembled nucleotides by the estimated genome size (i.e., 5 Mb).

Appendix 1 Table 2. Genomic characterization of Shiga toxin–producing *Escherichia coli* strains with extraintestinal pathogenic *E. coli*–associated virulence genes, the Netherlands and Italy, 2000–2019*

			Gene									
Strain	Serotype	ST (phylo-group)	*stx*	*eae*	*hlyF*	*ompT*	*iro BCDEN*	*iss*	*ehxa*	Origin	Patient diagnosis	Year (country)
ED0448	O186:H2	301 (B1)	stx_{2a}	*eae-ξ*	+	+	+	+	+	H	HUS	2000 (IT)
ED0463B	O186:H2	301 (B1)	stx_{2a}	*eae-ξ*	+	+	+	+	+	Beef liver	NA	2001 (IT)
ED0655	O80:H2	301 (B1)	stx_{2a}	*eae-ξ*	+	+	+	+	+	H	HUS	2007 (IT)
ED0656	O186:H2	301 (B1)	stx_{2a}	*eae-ξ*	+	+	+	+	+	H	HUS	2007 (IT)
ED0696	O80:H2	301 (B1)	stx_{2a}	*eae-ξ*	+	+	+	+	+	H	HUS	2009 (IT)
ED0812	O80:H2	301 (B1)	stx_{2a}	*eae-ξ*	+	+	+	+	+	H	HC	2011 (IT)
ED0813	O80:H2	301 (B1)	stx_{2a}	*eae-ξ*	+	+	+	+	+	H	HC	2011 (IT)
ED0840	O80:H2	301 (B1)	stx_{2a}	*eae-ξ*	+	+	+	+	+	H	HUS	2012 (IT)
ED0884	O80:H2	301 (B1)	stx_{2a}	*eae-ξ*	+	+	+	+	+	H	HC	2013 (IT)
ED0918	O80:H2	301 (B1)	stx_{2a}	*eae-ξ*	+	+	+	+	+	H	HUS	2013 (IT)
ED1000	O80:H2	301 (B1)	stx_{2a}	*eae-ξ*	+	+	+	+	+	H	HUS	2014 (IT)
ED1001	O80:H2	301 (B1)	stx_{2a}	*eae-ξ*	+	+	+	+	+	H	HUS	2014 (IT)
ED1029	O80:H2	301 (B1)	stx_{2a}	*eae-ξ*	+	+	+	+	+	H	HUS	2015 (IT)
ED1049	O80:H2	301 (B1)	stx_{2a}	*eae-ξ*	+	+	+	+	+	H	NA	2015 (IT)
ED1152	O80:H2	301 (B1)	stx_{2a}	*eae-ξ*	+	+	+	+	+	H	HC	2016 (IT)
ED1213	O80:H2	301 (B1)	stx_{2a}	*eae-ξ*	+	+	+	+	+	H	HUS	2017 (IT)
ED1232	O80:H2	301 (B1)	stx_{2a}	*eae-ξ*	+	+	+	+	+	H	HC	2017 (IT)
ED1269	O80:H2	301 (B1)	stx_{2a}	*eae-ξ*	+	+	+	+	+	H	NA	2018 (IT)
ED1273	O80:H2	301 (B1)	stx_{2a}	*eae-ξ*	+	+	+	+	+	H	NA	2018 (IT)
ED1283	O26:H11	21(B1)	stx_{2a}	*eae-β1*	+	+	+	+	+	H	HUS	2018 (IT)
ED1284	O26:H11	21(B1)	stx_{2a}	*eae-β1*	+	+	+	+	+	H	HUS	2018 (IT)
ED1304	O80:H2	301 (B1)	stx_{2a}	*eae-ξ*	+	+	+	+	+	H	HUS	2018 (IT)
ED1308	O80:H2	301 (B1)	stx_{2a}	*eae-ξ*	+	+	+	+	+	H	HUS	2018 (IT)
ED1319	O80:H2	301 (B1)	stx_{2d}	*eae-ξ*	+	+	+	+	+	H	HUS	2018 (IT)
ED1374	O80:H2	301 (B1)	stx_{2a}	*eae-ξ*	+	+	+	+	+	Raw bovine milk	NA	2019 (IT)
ED1381	O80:H2	301 (B1)	stx_{2a}	*eae-ξ*	+	+	+	+	+	H	HUS	2019 (IT)
ED1382	O80:H2	301 (B1)	stx_{2a}	*eae-ξ*	+	+	+	+	+	H	HUS	2019 (IT)
ED1386	O80:H2	301 (B1)	stx_{2a}	*eae-ξ*	+	+	+	–	+	H	HUS	2019 (IT)
EF0453	O80:H2	301 (B1)	stx_{2f}	*eae-ξ*	+	+	+	+	+	H	HUS	2013 (IT)
EF0475	O55:H9	301 (B1)	stx_{2f}	*eae-ξ*	+	+	+	+	+	H	HUS	2014 (IT)
NL1700223	O8:H9	88(Un)	stx_{2e}	–	+	+	+	+	–	H	NA	2017 (NL)
NL1700566	O152:H8	13(B1)	stx_{1a}	–	+	+	+	+	–	H	NA	2017 (NL)
NL1700800	O80:H2	301 (B1)	stx_{2a}	*eae-ξ*	+	+	+	+	+	H	NA	2017 (NL)
NL1700844	O80:H2	301 (B1)	stx_{2a}	*eae-ξ*	+	+	+	+	+	H	HC	2017 (NL)
NL1701143	O45:H2	301 (B1)	stx_{2a}	*eae-ξ*	+	+	+	+	+	H	NA	2017 (NL)
NL1701173	O80:H2	301 (B1)	stx_{2d}	*eae-ξ*	+	+	+	+	+	H	NA	2017 (NL)
NL1701358	O115:H52	8691 (B2)	stx_{2f}	*eae-λ3*	+	+	–	+	–	H, hosp.	D	2017 (NL)
NL1701474	O152:H8	13(B1)	stx_{1a}	–	+	+	+	+	–	H	NA	2017 (NL)
NL1701620	O8:H9	767 (B1)	stx_{2e}	–	+	+	+	+	–	H, hosp.	D	2017 (NL)
NL1800002	O80:H2	301 (B1)	stx_{2d}	*eae-ξ*	+	+	+	+	+	H	NA	2018 (NL)
NL1800025	O48:H45	656 (B1)	stx_{2b}	–	+	+	+	+	–	H	NA	2018 (NL)
NL1800037	O152:H8	13(B1)	stx_{1a}	–	+	+	+	+	–	H	NA	2018 (NL)
NL1800064	O80:H2	301 (B1)	stx_{2d}	*eae-ξ*	+	+	+	+	+	H, hosp.	HC	2018 (NL)
NL1800080	O80:H2	301 (B1)	stx_{2d}	*eae-ξ*	+	+	+	+	+	H	NA	2018 (NL)
NL1800418	O45:H2	301 (B1)	stx_{2a}	*eae-ξ*	+	+	+	+	+	H, hosp.	HC	2018 (NL)
NL1800531	O80:H2	301 (B1)	stx_{2f}	*eae-ξ*	+	+	+	+	+	H	D	2018 (NL)
NL1800673	O8:H9	88(Un)	stx_{2e}	–	+	+	+	+	–	H	D	2018 (NL)
NL1800717	O8:H9	88(Un)	stx_{2e}	–	+	+	+	+	–	H	NA	2018 (NL)
NL1800739	O80:H2	301 (B1)	stx_{2a}	*eae-ξ*	+	+	+	+	+	H	D	2018 (NL)
NL1800740	O45:H2	301 (B1)	stx_{2a}	*eae-ξ*	+	+	+	+	+	H	NA	2018 (NL)

Strain	Serotype	ST (phylo-group)	Gene							Origin	Patient diagnosis	Year (country)
			stx	*eae*	*hlyF*	*ompT*	*iro BCDEN*	*iss*	*ehxa*			
NL1900008	O91:H14	33(B1)	stx_{1a}	–	+	+	+	+	–	H	NA	2019 (NL)
NL1900697	O45:H2	301 (B1)	stx_{2a}	*eae-ξ*	+	+	+	+	+	H	D	2019 (NL)
NL1900824	O80:H2	301 (B1)	stx_{2d}	*eae-ξ*	+	+	+	+	+	H, hosp.	D	2019 (NL)

*D, diarrhea; H, human; HC, hemorrhagic colitis; HUS, hemolytic uremic syndrome; hosp., hospitalized; IT, Italy; NA, not available; NL, the Netherlands; ST, sequence type; Un, unknown; +, positive; –, negative.

Appendix 1 Table 3. Antimicrobial resistance genes associated with pR444_A plasmid that were identified in Shiga toxin–producing *Escherichia coli* strains with extraintestinal pathogenic *E. coli*–associated virulence genes, the Netherlands and Italy, 2000–2019*

Strain	Serotype	Sequence type	*stx*	AMR genes						
				aph(3')-I	*ant(3")-I*	*dfrA*	*str*	*sul*	bla_{TEM}	*tet*
ED0448	O186:H2	301	stx_{2a}	*aph(3')-Ia*	–	–	*strAB*	*sul2*	bla_{TEM-1}	*tet*(A)
ED0463B	O186:H2	301	stx_{2a}	*aph(3')-Ia*	–	*dfrA1*	*strAB*	*sul2*	bla_{TEM-1}	*tet*(A)
ED0655	O80:H2	301	stx_{2a}	*aph(3')-Ia*	–	–	*strAB*	*sul2*	bla_{TEM-1}	*tet*(A)
ED0656	O186:H2	301	stx_{2a}	*aph(3')-Ia*	–	*dfrA1*	*strAB*	*sul2*	bla_{TEM-1}	–
ED0696	O80:H2	301	stx_{2a}	*aph(3')-Ia*	–	–	*strAB*	*sul2*	bla_{TEM-1}	*tet*(A)
ED0812	O80:H2	301	stx_{2a}	–	*ant(3")-Ia*	–	–	*sul2*	–	*tet*(A)
ED0813	O80:H2	301	stx_{2a}	–	*ant(3")-Ia*	–	–	*sul2*	–	*tet*(A)
ED0840	O80:H2	301	stx_{2a}	*aph(3')-Ia*	–	–	*strAB*	*sul2*	bla_{TEM-1}	*tet*(A)
ED0884	O80:H2	301	stx_{2a}	*aph(3')-Ia*	–	–	*strAB*	*sul2*	bla_{TEM-1}	*tet*(A)
ED0918	O80:H2	301	stx_{2a}	*aph(3')-Ia*	–	–	*strAB*	*sul2*	bla_{TEM-1}	*tet*(A)
ED1000	O80:H2	301	stx_{2a}	*aph(3')-Ia*	–	–	*strAB*	*sul2*	bla_{TEM-1}	*tet*(A)
ED1001	O80:H2	301	stx_{2a}	*aph(3')-Ia*	–	–	*strAB*	*sul2*	bla_{TEM-1}	*tet*(A)
ED1029	O80:H2	301	stx_{2a}	*aph(3')-Ia*	–	–	*strAB*	*sul2*	–	*tet*(A)
ED1049	O80:H2	301	stx_{2a}	*aph(3')-Ia*	–	–	*strAB*	*sul2*	bla_{TEM-1}	*tet*(A)
ED1152	O80:H2	301	stx_{2a}	*aph(3')-Ia*	–	*dfrA17*	*strAB*	*sul2*	–	*tet*(A)
ED1213	O80:H2	301	stx_{2a}	–	*ant(3")-Ia*	–	–	*sul2*	–	*tet*(A)
ED1232	O80:H2	301	stx_{2a}	*aph(3")-Ib*	–	–	*strAB*	*sul2*	–	–
ED1269	O80:H2	301	stx_{2a}	*aph(3')-Ia*	–	–	*strAB*	*sul2*	bla_{TEM-1}	*tet*(A)
ED1273	O80:H2	301	stx_{2a}	–	*ant(3")-Ia*	–	–	*sul2*	–	*tet*(A)
ED1283	O26:H11	21	stx_{2a}	*aph(3")-Ib*	–	*dfrA1*	*strAB*	*sul2*	bla_{TEM-1}	*tet*(A)
ED1284	O26:H11	21	stx_{2a}	*aph(3")-Ib*	–	*dfrA1*	*strAB*	*sul2*	bla_{TEM-1}	*tet*(A)
ED1304	O80:H2	301	$stx2_{a}$	*aph(3')-Ia*	–	–	*strAB*	*sul2*	bla_{TEM-1}	*tet*(A)
ED1308	O80:H2	301	stx_{2a}	*aph(3')-Ia*	–	–	*strAB*	*sul2*	bla_{TEM-1}	*tet*(A)
ED1319	O80:H2	301	stx_{2d}	*aph(3')-Ia*	–	*dfrA5*	*strAB*	*sul2*	bla_{TEM-1}	*tet*(C)
ED1374	O80:H2	301	stx_{2a}	*aph(3')-Ia*	–	–	*strAB*	*sul2*	bla_{TEM-1}	*tet*(A)
ED1381	O80:H2	301	stx_{2a}	*aph(3')-Ia*	–	–	*strAB*	*sul2*	bla_{TEM-1}	*tet*(A)
ED1382	O80:H2	301	stx_{2a}	–	*ant(3")-Ia*	–	–	*sul2*	–	*tet*(A)
ED1386	O80:H2	301	stx_{2a}	*aph(3')-Ia*	–	–	*strAB*	*sul1*	bla_{TEM-1}	*tet*(A)
EF0453	O80:H2	301	stx_{2f}	*aph(3')-Ia*	–	–	*strAB*	*sul2*	bla_{TEM-1}	*tet*(A)
EF0475	O55:H9	301	stx_{2f}	*aph(3")-Ib*	–	–	*strAB*	*sul2*	bla_{TEM-1}	–
NL1700223	O8:H9	88	stx_{2e}	–	–	*dfrA5*	–	–	–	–
NL1700566	O152:H8	13	stx_{1a}	–	–	–	–	–	–	–
NL1700800	O80:H2	301	stx_{2a}	*aph(3')-Ia*	–	–	*strAB*	*sul2*	–	*tet*(A)
NL1700844	O80:H2	301	stx_{2a}	*aph(3')-Ia*	–	*dfrA5*	*strAB*	*sul2*	bla_{TEM-1}	*tet*(A)
NL1701143	O45:H2	301	stx_{2a}	*aph(3')-Ia*	–	–	*strAB*	*sul2*	bla_{TEM-1}	*tet*(A)
NL1701173	O80:H2	301	stx_{2d}	*aph(3')-Ia*	–	*dfrA5*	*strAB*	*sul2*	bla_{TEM-1}	–
NL1701358	O115:H52	8691	stx_{2f}	–	–	–	–	–	–	–
NL1701474	O152:H8	13	stx_{1a}	–	–	–	–	–	–	–
NL1701620	O8:H9	767	stx_{2e}	–	–	*dfrA17*	–	–	bla_{TEM-1}	*tet*(A)
NL1800002	O80:H2	301	stx_{2d}	*aph(3')-Ia*	–	*dfrA5*	*strAB*	*sul2*	bla_{TEM-1}	*tet*(A)
NL1800025	O48:H45	656	stx_{2b}	*aph(3")-Ib*	–	–	*strAB*	*sul2*	–	*tet*(A)
NL1800037	O152:H8	13	stx_{1a}	–	–	–	–	–	–	–
NL1800064	O80:H2	301	stx_{2d}	*aph(3')-Ia*	–	*dfrA5*	*strAB*	*sul2*	bla_{TEM-1}	–
NL1800080	O80:H2	301	stx_{2d}	*aph(3')-Ia*	–	*dfrA5*	*strAB*	*sul2*	bla_{TEM-1}	–
NL1800418	O45:H2	301	stx_{2a}	*aph(3')-Ia*	–	–	*strAB*	*sul2*	–	*tet*(A)
NL1800531	O80:H2	301	stx_{2f}	*aph(3')-Ia*	–	–	*strAB*	*sul2*	bla_{TEM-1}	*tet*(A)
NL1800673	O8:H9	88	stx_{2e}	*aph(3")-Ib*	–	*dfrA5*	*strAB*	*sul2*	bla_{TEM-1}	–
NL1800717	O8:H9	88	stx_{2e}	*aph(3")-Ib*	–	*dfrA5*	*strAB*	*sul2*	bla_{TEM-1}	–
NL1800739	O80:H2	301	stx_{2a}	–	–	–	–	*sul2*	–	*tet*(A)
NL1800740	O45:H2	301	stx_{2a}	*aph(3')-Ia*	–	–	*strAB*	*sul2*	bla_{TEM-1}	*tet*(A)
NL1900008	O91:H14	33	stx_{1a}	–	–	–	–	–	–	*tet*(A)
NL1900697	O45:H2	301	stx_{2a}	*aph(3')-Ia*	–	–	*strAB*	*sul2*	bla_{TEM-1}	*tet*(A)
NL1900824	O80:H2	301	stx_{2d}	*aph(3')-Ia*	–	–	*strAB*	*sul2*	bla_{TEM-1}	–

**aph(3')-I* and *ant(3")-I* encode resistance to aminoglycosides; *dfrA* encodes resistance to trimethoprim; *sul* encodes resistance to sulfonamides; bla_{TEM} encodes resistance to β-lactams; *tet* encodes resistance to tetracyclines.

Appendix 1 Table 4. Main characteristics of the 50 *Escherichia coli* genomes downloaded from GenBank and RefSeq*

Strain	Accession no.	Serotype	Sequence type (Phylogroup)	Source	Country (city or state)	Year
2013C-4991	GCF_003018815.1	O80:H2	301 (B1)	Human, NA	NA	2013
31707	GCF_003122965.2	O80:H2	301 (B1)	Human, HUS	France (Paris)	2017
364060–17	GCF_003028275.1	O80:H2	301 (B1)	NA	Switzerland (Zurich)	2017
364061–17	GCF_003028045.1	O80:H2	301 (B1)	NA	Switzerland (Zurich)	2017
364062–17	GCF_003028145.1	O80:H2	301 (B1)	NA	Switzerland (Zurich)	2017
364064–17	GCF_003028245.1	O80:H2	301 (B1)	NA	Switzerland (Zurich)	2017
364068–17	GCF_003027955.1	O80:H2	301 (B1)	NA	Switzerland (Zurich)	2017
364069–17	GCF_003028095.1	O80:H2	301 (B1)	NA	Switzerland (Zurich)	2017
364073–17	GCF_003027915.1	O80:H2	301 (B1)	NA	Switzerland (Zurich)	2017
364075–17	GCF_003028075.1	O80:H2	301 (B1)	NA	Switzerland (Zurich)	2017
364077–17	GCF_003028035.1	O80:H2	301 (B1)	NA	Switzerland (Zurich)	2017
364082–17	GCF_003027965.1	O80:H2	301 (B1)	NA	Switzerland (Zurich)	2017
36549	GCF_003122855.2	O80:H2	301 (B1)	Human, HUS	France (Paris)	2017
37619	GCF_003123295.1	O80:H2	301 (B1)	Human, HUS	France (Paris)	2017
40963	GCF_003123255.1	O80:H2	301 (B1)	Human, HUS	France (Paris)	2017
CB12623	GCF_003123165.2	O80:H2	301 (B1)	Human, HUS	Switzerland (Berne)	2017
CM15–2	GCF_004664645.1	O8:H16	2217 (B1)	Ground beef	Argentina (Tandil)	1998
EH1752	GCF_013413115.1	O80:H2	301 (B1)	Human, D	Belgium	2008
EH1764	GCF_013413035.1	O80:H2	301 (B1)	Human, D	Belgium	2008
EH2262	GCF_013413055.1	O80:H2	301 (B1)	Human, D	Belgium	2013
EH2400	GCF_013413045.1	O80:H2	301 (B1)	Human, D	Belgium	2014
EH2436	GCF_013413065.1	O80:H2	301 (B1)	Human, NA	Belgium	2014
EH2644	GCF_013412965.1	O80:H2	301 (B1)	Human, HUS	Belgium	2015
EH2786	GCF_013413335.1	O80:H2	301 (B1)	Human, NA	Belgium	2016
EH2808	GCF_013412955.1	O80:H2	301 (B1)	Human, HUS	Belgium	2016
EH2882	GCF_013413375.1	O80:H2	301 (B1)	Calf, D	Belgium	1987
EH3138	GCF_013412915.1	O80:H2	301 (B1)	Human, HC	Belgium	2018
EH3154	GCF_013413215.1	O80:H2	301 (B1)	Calf, Ent	Belgium	2018
EH3155	GCF_013413175.1	O80:H2	301 (B1)	Calf, Ent	Belgium	2018
EH3160	GCF_013413135.1	O80:H2	301 (B1)	Calf, Ent	Belgium	2018
EH3168	GCF_013412945.1	O80:H2	301 (B1)	Human, HC	Belgium	2018
EH3172	GCF_013412895.1	O80:H2	301 (B1)	Human, D	Belgium	2018
EH3173	GCF_013412825.1	O80:H2	301 (B1)	Human, NA	Belgium	2018
EH3180	GCF_013412835.1	O80:H2	301 (B1)	Human, NA	Belgium	2018
EH3205	GCF_013412845.1	O80:H2	301 (B1)	Human, D	Belgium	2019
EH3212	GCF_013412765.1	O80:H2	301 (B1)	Human, HUS	Belgium	2019
EH3257	GCF_013412765.1	O80:H2	301 (B1)	Human, HUS†	Belgium	2019
EH3278	GCF_013412755.1	O80:H2	301 (B1)	Human, HUS	Belgium	2019
EH3307	GCF_013413315.1	O80:H2	301 (B1)	Calf, D	Belgium	2016
EH3310	GCF_013413285.1	O80:H2	301 (B1)	Calf, D	Belgium	2016
EH3311	GCF_013413275.1	O80:H2	301 (B1)	Calf, D	Belgium	2016
EH3315	GCF_013413235.1	O80:H2	301 (B1)	Calf, D	Belgium	2017
EH3320	GCF_013413165.1	O80:H2	301 (B1)	Calf, D	Belgium	2017
EH3338	GCF_013413145.1	O80:H2	301 (B1)	Calf, Sept	Belgium	2018
H39–78	GCF_003123395.1	O80:H2	301 (B1)	Cattle, NA	France (Lyon)	2017
LMFS-V-JF-008	GCA_014451005.1	O103:H2	17 (B1)	Surface water	Canada (Sumas Prairie)	2015
LMFS-V-JF-010	GCF_014050405.1	O109:H5	647 (B2)	Surface water	Canada (Sumas Prairie)	2015
NGE.clc	GCF_001191215.1	O45:H16	2217 (B1)	Cow, NA	USA (Kansas)	2001
OLC1061	GCF_002134035.1	O128ab:H2	25 (B1)	NA	Canada	2012
RM11911	GCF_008761495.2	O45:H16	2217 (B1)	Water	USA (California)	2010

*RefSeq, www.ncbi.nlm.nih.gov/RefSeq. All strains were positive for *stx* and *hlyF* genes. HC, hemorrhagic colitis; HUS, hemolytic uremic syndrome; NA, not available; D, diarrhea;
Sept, septicemia; Ent, enteritis.
†Fatal disease.

Appendix 1 Table 5. Statistics output of core genome multilocus sequence typing analysis of *hlyF*-positive Shiga toxin–producing *Escherichia coli* strains used in this study*

Genome	Exact match	Allele inferred	Locus not found	Possible locus on tip	Noninformative paralogous hits	Alleles larger than mode	Alleles smaller than mode
2013C-4991	2341	0	6	1	6	0	6
31707	2342	0	8	0	7	0	3
364060–17	2339	0	9	6	5	0	1
364061–17	2348	0	4	1	7	0	0
364062–17	2348	0	4	0	7	0	1
364064–17	2348	0	5	0	7	0	0
364068–17	2350	0	4	0	6	0	0
364069–17	2349	0	3	1	7	0	0
364073–17	2349	0	2	0	7	0	2
364075–17	2350	0	3	0	7	0	0
364077–17	2349	0	4	0	7	0	0
364082–17	2349	0	3	0	7	0	1
36549	2348	0	3	2	7	0	0
37619	2306	0	38	9	7	0	0
40963	2294	0	39	19	7	1	0
CB12623	2344	0	5	4	5	0	2
CM15–2	2352	0	4	1	1	0	2
ED0448	2344	0	6	1	5	0	4
ED0463B	2338	0	12	3	5	0	2
ED0655	2346	0	6	0	6	0	2
ED0656	2339	0	12	2	4	0	3
ED0696	2346	0	6	1	6	0	1
ED0812	2336	0	9	3	6	0	6
ED0813	2344	0	6	0	7	0	3
ED0840	2342	0	6	1	7	0	4
ED0867	2338	0	12	2	6	0	2
ED0884	2344	0	4	2	6	0	4
ED0918	2350	0	3	0	4	0	3
ED1000	2343	0	7	0	6	0	4
ED1001	2338	0	8	2	7	0	5
ED1029	2347	0	6	0	6	0	1
ED1049	2348	0	4	0	7	0	1
ED1152	2344	0	5	2	7	0	2
ED1213	2342	0	6	2	6	0	4
ED1232	2344	0	6	3	5	0	2
ED1269	2303	0	47	0	7	1	2
ED1273	2346	0	4	0	7	0	3
ED1283	2348	0	0	5	5	0	2
ED1284	2347	0	3	4	4	0	2
ED1301	2343	0	7	0	7	1	2
ED1304	2341	0	10	3	4	0	2
ED1308	2339	0	14	1	5	0	1
ED1319	2336	0	8	0	7	0	9
ED1374	2338	0	14	1	5	0	2
ED1381	2341	0	4	2	7	0	6
ED1382	2346	0	6	2	5	0	1
ED1386	2346	0	5	0	5	0	4
EF0453	2342	0	8	2	6	0	2
EF0475	2342	0	6	1	6	1	4
EH1752	2351	0	3	0	6	0	0
EH1764	2350	0	3	0	7	0	0
EH2262	2351	0	2	0	6	0	1
EH2400	2350	0	3	0	7	0	0
EH2436	2346	0	7	0	6	0	1
EH2644	2349	0	3	0	7	0	1
EH2786	2351	0	2	1	6	0	0
EH2808	2346	0	7	0	6	0	1
EH2882	2350	0	3	0	5	0	2
EH3138	2351	0	2	0	6	0	1
EH3154	2348	0	6	0	5	0	1
EH3155	2350	0	4	0	6	0	0
EH3160	2350	0	4	0	6	0	0
EH3168	2349	0	4	1	6	0	0
EH3172	2349	0	4	0	7	0	0
EH3173	2350	0	3	0	7	0	0
EH3180	2350	0	3	0	7	0	0

Genome	Exact match	Allele inferred	Locus not found	Possible locus on tip	Noninformative paralogous hits	Alleles larger than mode	Alleles smaller than mode
EH3205	2344	0	11	0	5	0	0
EH3212	2349	0	3	1	7	0	0
EH3257	2349	0	3	1	7	0	0
EH3278	2349	0	3	1	7	0	0
EH3307	2339	0	15	0	5	0	1
EH3310	2349	0	3	0	7	0	1
EH3311	2342	0	9	0	7	1	1
EH3315	2342	0	9	0	7	1	1
EH3320	2349	0	3	0	7	0	1
EH3338	2350	0	4	0	6	0	0
H39–78	2347	0	4	1	7	0	1
LMFS-V-JF-008	2354	0	0	0	6	0	0
LMFS-V-JF-010	2351	0	6	0	1	0	2
NGE.clc	2343	0	10	1	3	0	3
NL1700223	2356	0	2	0	2	0	0
NL1700566	2348	0	4	3	2	1	2
NL1700800	2353	0	2	0	5	0	0
NL1700844	2349	0	4	0	7	0	0
NL1701143	2349	0	3	1	6	0	1
NL1701173	2349	0	5	0	6	0	0
NL1701358	2271	0	80	0	4	1	4
NL1701474	2350	0	4	2	2	1	1
NL1701620	2352	0	4	1	3	0	0
NL1800002	2345	0	8	1	6	0	0
NL1800025	2338	0	14	2	2	0	4
NL1800037	2350	0	4	2	2	1	1
NL1800064	2350	0	4	0	6	0	0
NL1800080	2350	0	4	0	6	0	0
NL1800418	2352	0	2	1	5	0	0
NL1800531	2349	0	4	0	7	0	0
NL1800673	2352	0	5	0	2	0	1
NL1800717	2352	0	5	0	2	0	1
NL1800739	2352	0	2	0	6	0	0
NL1800740	2352	0	2	1	5	0	0
NL1900008	2349	0	7	0	2	0	2
NL1900697	2350	0	3	1	5	0	1
NL1900824	2349	0	4	0	6	0	1
OLC1061	2351	0	5	1	3	0	0
RDEx444	2338	0	10	0	7	0	5
RM11911	2341	0	9	0	2	2	6

*Analysis conducted with the chewBBACA tool (https://doi.org/10.1099/mgen.0.000166).
